# Quantitative analysis of facet growth during directional solidification of salol

**DOI:** 10.1107/S2052520626001769

**Published:** 2026-03-05

**Authors:** Anassya Raad, Nathalie Bergeon, Nathalie Mangelinck-Noël, Fatima L. Mota

**Affiliations:** a Aix Marseille Univ, Université de Toulon CNRS, IM2NP Marseille France; Siberian Branch of Russian Academy of Science, Russian Federation

**Keywords:** directional solidification, *in situ* observation, transparent systems, thin sample, facet growth

## Abstract

The growth dynamics of faceted materials exhibit slow attachment kinetics, strong solid–liquid anisotropy, and specific kinetic coefficients. The formation of structures, facet dynamics and defects during growth strongly influence the properties of the resulting materials. Using the transparent model material salol and the method of controlled directional solidification, this work aims to provide a deeper understanding of the formation of structures in faceted materials, which remain insufficiently understood. The study reveals that after an initial transient period, the solid–liquid interface stabilizes, with its position linked to the interface undercooling. Facet size heterogeneity is characterized with a tendency to larger facets for lower thermal gradients. A methodology is developed to exploit experimentally measured angles into a three-dimensional angle in the crystals, allowing the identification of the crystallographic nature of the facets. Despite random and unknown initial orientations, the interface is predominantly bounded by {111} planes. Facet velocities are analyzed, and deviations from theoretical predictions are attributed to competitive growth processes. The analysis of facet velocities as a function of undercooling suggests a growth mechanism primarily controlled by two-dimensional nucleation. It is shown that the experimental data relating the growth rate of {111} facets to the undercooling correspond to bi-dimensional kinetic growth law in most cases. Only a few data points can be related to a kinetic growth law driven by screw dislocation emerging at the {111} facet surface.

## Abstract 2

This study offers a comprehensive analysis of facet growth during directional solidification of salol, focusing on the dynamic evolution of the solid–liquid interface. A novel strategy was developed to identify the crystallographic nature of the observed facets, whose growth is mainly governed by two-dimensional nucleation, with deviations attributed to competitive growth processes.

## 1. Introduction

Solidification is the process by which a liquid transforms into a solid, as observed in the formation of ice, the freezing of solder in electrical circuits, or metal casting in industrial manufacturing. Solidification has been of interest to humans since the Bronze Age and continues to be a major focus in the modern era. Even today, researchers and industry professionals seek to understand and control the morphology of the solid–liquid interface, as it plays a critical role in determining the final material properties and ensuring high-quality products (Dantzig & Rappaz, 2016 ▸; Flemings, 1974 ▸; Kurz & Fisher, 1998 ▸). During solidification from the melt, the solid–liquid interface morphology depends on both the material physical properties and the processing parameters. To study these effects, directional solidification is particularly effective, as it allows precise control of process conditions. Rough interfaces at the atomic scale are representative of materials with low surface energy anisotropy, such as metallic materials. The attachment of atoms to the interface is easy and instantaneous, inducing rapid growth kinetics and morphology controlled by temperature and chemical species diffusion phenomena. Faceted interfaces at the macroscopic scale are a common morphology observed during the solidification of many materials, such as semiconductors and quasicrystals (Deville, 2013 ▸; Fujiwara *et al.*, 2022 ▸; Lenart *et al.*, 2012 ▸; Riberi-Béridot *et al.*, 2019 ▸; Tsoutsouva *et al.*, 2016 ▸; Ouaddah *et al.*, 2020 ▸; Becker *et al.*, 2019 ▸). In such cases, the atoms attachment from the liquid at the interface is anisotropic and relatively slow, and its effect is dominant during growth. As a result, crystals exhibit smooth surfaces at the atomic scale.

Numerical simulations are commonly employed to understand and analyze microstructure formation during solidification, with the ultimate goal of optimizing process parameters. While metallic alloys and their transparent analogs have been extensively studied and modeled in the context of solidification (Kurz *et al.*, 2019 ▸; Kurz *et al.*, 2021 ▸; Mota *et al.*, 2015 ▸; Mota *et al.*, 2023 ▸; Mota *et al.*, 2021 ▸; Song *et al.*, 2023 ▸; Song *et al.*, 2018 ▸), the growth dynamics of faceted materials remain less explored and are still not well understood. Modeling faceted growth is particularly challenging due to the lack of quantitative data on the properties of the solid–liquid interface, which hinders the development of accurate models and leads to largely qualitative interpretations of the few existing experimental results (Braik *et al.*, 2024 ▸).

A clear understanding of the dynamics of faceted morphologies remains a significant challenge and requires key insights that can only be obtained through *in situ* and real time observations of the process. This can be achieved through model experiments using transparent organic materials (Huang & Wang, 2012 ▸; Jackson & Hunt, 1965 ▸), which offer the advantages such as transparency to visible light and relatively low melting points, typically below 373 K. Hunt *et al.* (1966 ▸) made a significant breakthrough in studying transparent model alloys during solidification. However, less attention has been given to transparent faceting organic compounds. Shangguan & Hunt (1989 ▸; 1991 ▸; 1992 ▸) were among the pioneers to observe faceted growth *in situ* and study the underlying physical process and growth mechanisms using various organic materials, such as salol, thymol, and *o*-terphenyl. In the 1990s, Fabietti &Trivedi (1991 ▸; 1997*a* ▸; 1997*b* ▸) conducted detailed studies on the directional solidification of faceted materials to characterize growth mechanisms and explore the relationship between interface morphology and experimental conditions. Dey & Sekhar (1993*a* ▸; 1993*b* ▸) focused specifically on the growth of salol crystals, combining numerical and experimental approaches to determine the conditions for the onset of cracks during directional growth of macroscopically faceted crystals. Higashino *et al.* (1993 ▸) simultaneously measured growth rates, temperature profiles, and solute concentration in the vicinity of the solid–liquid interface. They found that the temperature distribution ahead of the interface is significantly influenced by the released latent heat. Later, Inatomi *et al.* (1997 ▸; 2008 ▸) studied the effect of gravity on temperature and concentration distributions in front of the interface. Several studies have since focused on the study of the formation and distribution of defects, which can significantly impact the quality and properties of the final crystal structure (Fabietti & Trivedi, 1997*a* ▸; Borzsonyi *et al.*, 2009 ▸; Fabietti & Trivedi, 1997*b* ▸; Raad *et al.*, 2025 ▸; Fabietti & Trivedi, 1991 ▸; Li *et al.*, 2022 ▸; Scheffen-Lauenroth *et al.*, 1981 ▸; Klapper, 1980 ▸).

In general, the normal growth velocity at the solid–liquid interface can be expressed as a function of undercooling. The kinetic attachment of atoms during faceted growth depends on the local crystallographic orientation, and the relationship between kinetic undercooling and velocity is determined by the growth mechanism (Flemings, 1974 ▸). When atoms or molecules are continuously added to the interface, it advances uniformly, leading to continuous growth. In this case, the growth velocity is directly proportional to the undercooling. However, in materials with high melting entropy atomically smooth, close-packed interfaces are formed preferentially. To advance such an atomically smooth interface in a direction normal to itself during crystal growth requires lateral growth of new layers. When critical nuclei form on the surface of a crystal and subsequently expand to create a new layer, lateral growth occurs via two-dimensional nucleation. Here, the relationship between the growth velocity and kinetic undercooling follows an inverse exponential form. Finally, when a screw dislocation emerges at the solid–liquid interface, creating a step on the crystal surface, lateral growth is accelerated and driven by this mechanism. The relationship between the growth velocity and kinetic undercooling follows a quadratic law (V is proportional to the square of the undercooling) in this case.

There is some debate regarding the nature of the growth mechanisms operating under different growth conditions. For example, concerning salol, although studies on its growth kinetics are numerous and long-standing, there is considerable scatter in the reported data (Cahn, 1960 ▸; Cahn *et al.*, 1964 ▸; Danilov & Malkin, 1954 ▸; Ie & Strickland-Constable, 1974 ▸; Jackson *et al.*, 1967 ▸; Jin *et al.*, 1990 ▸; Malkin, 1954 ▸; Morris *et al.*, 1968 ▸; Neumann & Micus, 1954 ▸; Podolinski, 1979 ▸; Pollatschek, 1929 ▸; Riveros, 1968 ▸). However, most of the growth rate-undercooling relationships have been measured only during the free growth of salol crystals, which primarily involve facets belonging to the {100}, {010}, {110}, {201}, {210} and {211} crystallographic planes. In general, these studies agree that, depending on the undercooling, there is a transition from lateral to continuous growth. Dey and Sekhar (1993*a* ▸) focused on the kinetics of {111} crystallographic planes during recalascent experiments. After being pulled at high velocity (100 µm s^−1^), the movement was stopped and the interface was allowed to recalesce. They found that at low undercoolings, growth follows a two-dimensional nucleation-controlled mechanism, while at higher undercoolings, the growth transitions to a continuous mode, with a change in the interface morphology. Fabietti & Trivedi (1991 ▸) were the first to use the directional solidification technique to obtain the response functions for the nonequilibrium conditions that exist at the interface during growth. Directional solidification studies have been carried out in the naphthalene–camphor system, and it was found that the mechanisms of planar interface instability and the subsequent morphological development of the interface depend significantly on the crystallographic orientation of the interface. The interface kinetic law is found to be exponential, indicating that the growth of the interface occurs through the process of nucleation of new layers.

The main objective of this work is to identify the crystallographic nature of the facets formed during the directional solidification of salol, starting from an initial seed with random orientation. The study also aims to derive the facet growth laws as a function of the undercooling. A series of experiments are carried out in a Bridgman type furnace with different thermal gradients and pulling velocities, each resulting in a highly faceted solid–liquid interface. This paper is organized as follows. Section 2 describes the experimental set-up and the procedures used, including the characterization of the crystals. Section 3 presents the results of the experimental observations and discusses the findings, which include the analysis of the solid–liquid interface movement, essential for characterizing facet undercooling, facet measurement and crystallographic indexing, and the analysis of facet dynamics to derive growth laws.

## 2. Experimental procedure

### 2.1. Crystal growth

**Figure 1:**
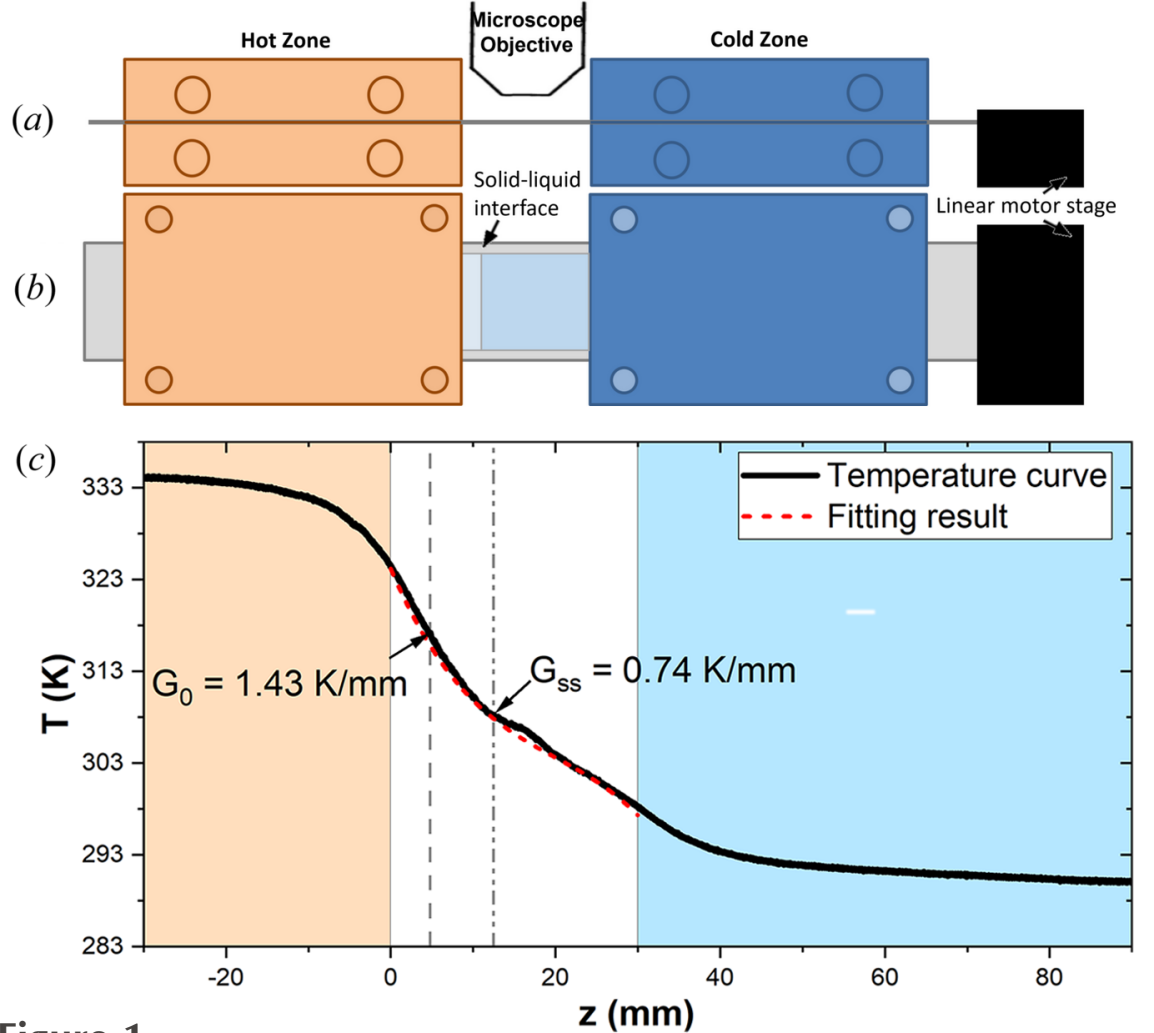
Schematic diagram of the Bridgman-type furnace seen from (*a*) the side and (*b*) the top. (*c*) Temperature profile obtained with a Type K thermocouple inside a salol sample at *V*_p_ = 10 µm s^−1^. The local thermal gradient is given at rest and steady-state positions, *G*_0_ and *G*_ss_, respectively.

In this work, salol crystals are grown using a two thermal zones horizontal Bridgman furnace (Raad *et al.*, 2025 ▸). The as-received salol (99%, Sigma-Aldrich) is purified by distillation before the experiments, and its purity is determined to be greater than 99.9% through melting point measurements and elemental analysis. The schematic diagram of the Bridgman furnace is shown in Figs. 1 ▸(*a*) and 1 ▸(*b*). A key factor for the investigations of growth laws is temperature, which is controlled in this case by adjusting the hot and cold zones temperatures in the furnace. Setting these temperatures establishes a thermal gradient *G*, which is measured prior to the growth experiments by a dedicated experimental protocol. For this measurement, an ultra-fine wire Type K thermocouple (0.8 mm diameter) is inserted into a sealed crucible filled with salol and connected to a thermocouple input module with ±0.080 V precision. A temperature curve as a function of position is obtained by moving the sample, along with the thermocouple, through the furnace at constant pulling velocity *V*_p_ [Fig. 1 ▸(*c*)]. The temperature data as function of the distance within the non-regulated zone are fitted to a cubic function. The derivative of the fitting curve is then used to obtain the local thermal gradient that, in the shown example, is not constant within the non-regulated zone. For imposed temperatures of 343 and 283 K in the hot and cold zones, respectively, with a 30 mm gap between the thermal zones, the salol interface at rest is positioned at 4.8 mm within the gap, corresponding to a thermal gradient *G*_0_ of 1.43 K mm^−1^. During pulling at 10 µm s^−1^, its steady-state position is at 12.5 mm, corresponding to a thermal gradient *G*_ss_ of 0.74 K mm^−1^. A different configuration is also used, where the thermal zones are closer together (gap of 11 mm), with imposed temperatures of 333 and 283 K in the hot and cold zones, respectively. In this case, the thermal gradient is constant within the non-regulated thermal zone. This procedure is repeated for different temperatures settings, gaps, and pulling velocities to determine the local thermal gradient for all the experimental conditions tested.

Salol is placed in a cell composed of two borosilicate glass slides (150 × 130 mm) separated by a 50 µm V-shaped Mylar spacer. The sample cell is placed on the Bridgman furnace and first melted at constant velocity until a very small amount of solid remains. A few millimetres are then solidified, and the sample is kept in temperature at this position overnight. This step is essential to ensure uniform temperature distribution, prevent thermal stresses, stabilize the growth front, promote monocrystalline growth as much as possible, minimize defects within the crystal, and prepare a controlled starting point for solidification (Kurz & Fisher, 1998 ▸; Glicksman, 2010 ▸). Then, directional solidification experiments are conducted under various growth conditions (*V*_p_ and *G*). The sample is moved across the thermal gradient using an ultra-precision linear motor stage and a universal motion controller. In all the experiments, the solid–liquid interface is photographed by a camera mounted on an optical microscope, at regular time intervals (in general every 2 s) throughout the solidification process, allowing for *in situ* and real time characterization of the interface morphology.

### 2.2. Crystal characterization

The crystal structure of salol is created on the basis of literature information in the software *VESTA*. *VESTA* (Visualization for Electronic and Structural Analysis) is a cross-platform program for 3D visualization and investigation of crystal structure data, volumetric data and crystal morphology data (https://jp-minerals.org/vesta/en/; de la Flor *et al.*, 2024 ▸; Momma & Izumi, 2011 ▸). Salol crystals have an orthorhombic structure of space group *Pcab*, with unit-cell parameters, measured by X-ray diffraction, of *a* = 23.50 Å, *b* = 11.31 Å and *c* = 8.10 Å, which are in good agreement with literature values (Bilgram *et al.*, 1982 ▸; Riveros, 1968 ▸; Flachsbart, 1957 ▸). The final positional parameters of carbon, oxygen and hydrogen atoms are also given by these authors. Then, based on the typical morphology of salol crystals reported in literature (Neuroth & Klapper, 1994 ▸; Wyrouboff, 1889 ▸), the main crystal faces are identified according to their Miller indices ({100}, {010}, {111}, {210} and {211}), and the remaining faces are automatically generated based on the corresponding symmetry. The outer boundaries of a macroscopic crystal, which may contain a huge number of unit cells, is created. Once the macroscopic crystal is built in *VESTA*, it can be rotated in any direction. The respective matrix that describes the rotation and orientation of the crystal structure in 3D space is available, and it defines how the crystal axes (*a*, *b*, *c*) are aligned relative to the coordinate system used for visualization.

## 3. Results and discussion

### 3.1. Solid–liquid interface movement

**Figure 2:**
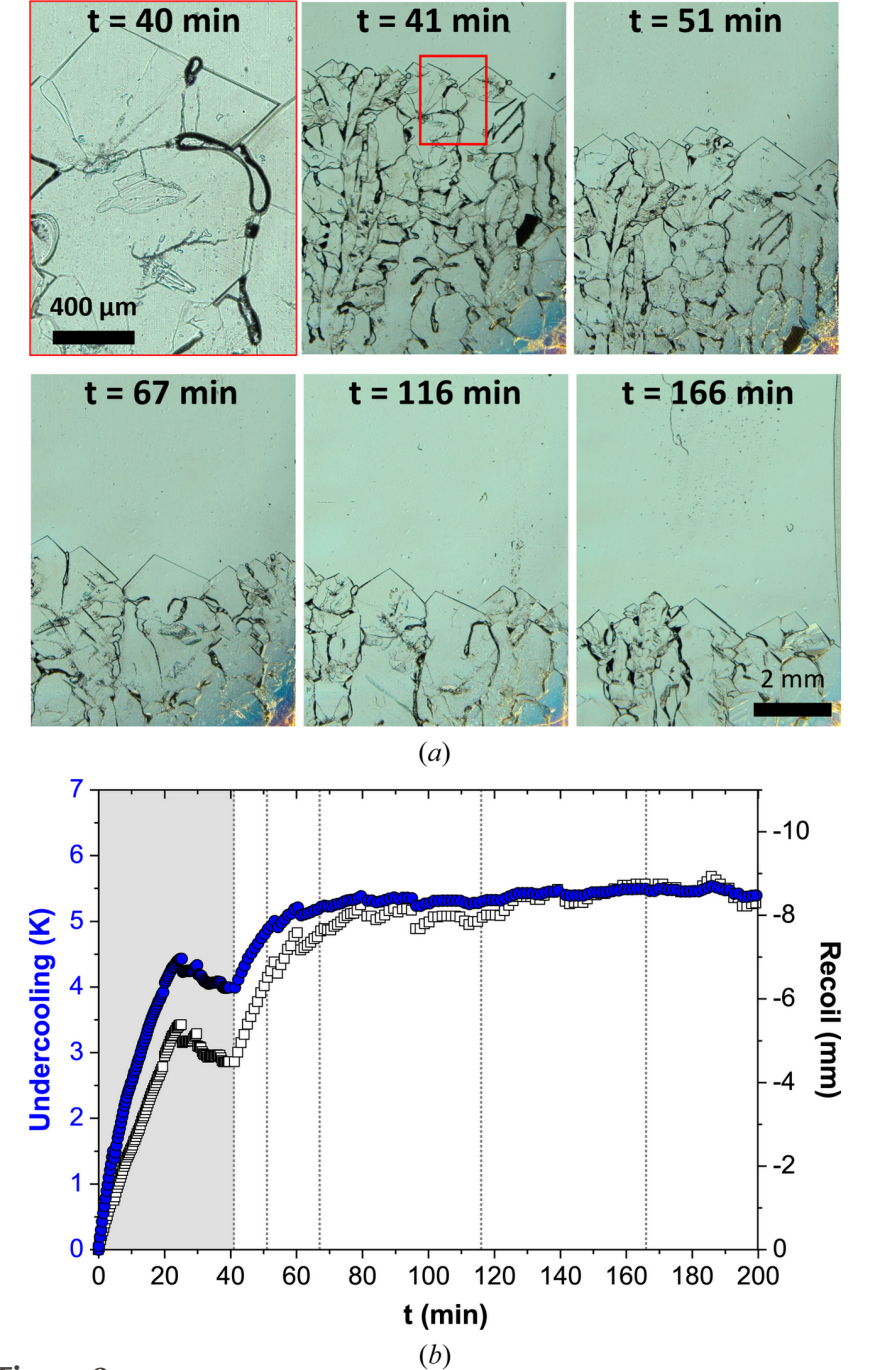
(*a*) Images of the solid–liquid interface of salol as function of time. (*b*) Evolution of the undercooling (blue circles, left *y* axis) and recoil (empty squares, right *y* axis) as function of time. The vertical dotted lines correspond to the times in (*a*). (*V*_p_ = 5 µm s^−1^, *G*_0_ = 1.3 K mm^−1^).

**Figure 3:**
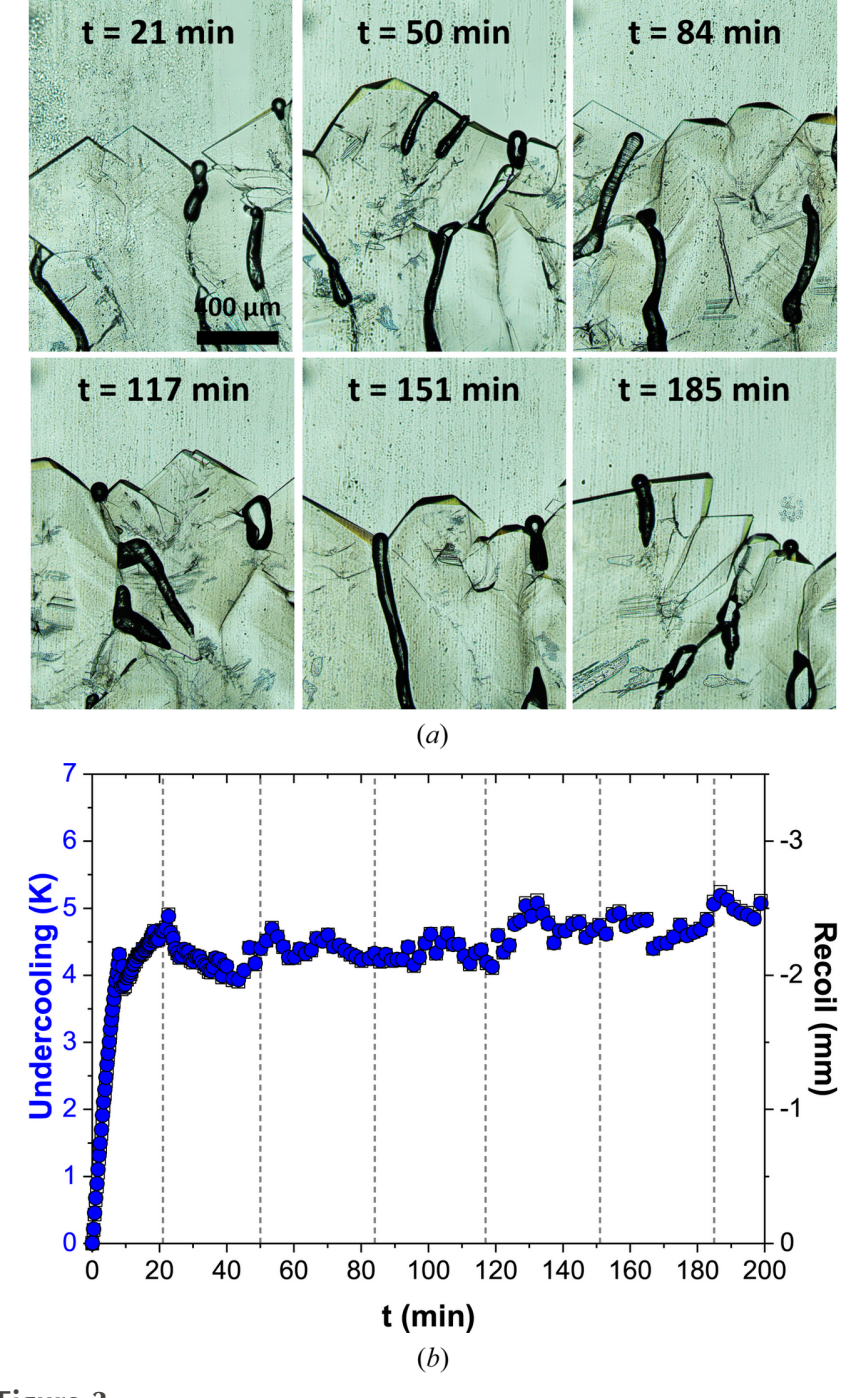
(*a*) Images of the solid–liquid interface of salol as function of time. (*b*) Evolution of the undercooling (blue circles, left *y* axis) and recoil (empty squares, right *y* axis) as function of time. The vertical dotted lines correspond to the times in (*a*). (*V*_p_ = 5 µm s^−1^, *G*_0_ = 2.1 K mm^−1^).

*In situ* and real-time observation during directional solidification enables continuous monitoring of the solid–liquid interface over time. Fig. 2 ▸(*a*) shows the evolution of the solid–liquid interface during a directional solidification experiment at *V*_p_ = 5 µm s^−1^ and *G*_0_ = 1.3 K mm^−1^. At 41 min, the microscope objective was switched to lower magnification to provide a broader view of the interface. Only one image at the initial magnification is shown (*t* = 40 min). The region observed prior to 41 min is highlighted by the red square in the image at that time. Throughout the experiment, a highly polycrystalline faceted structure is observed, exhibiting complex and dynamic growth at the interface. Similarly, Fig. 3 ▸(*a*) shows the evolution of the interface in an experiment with *V*_p_ = 5 µm s^−1^ and *G*_0_ = 2.1 K mm^−1^. The formation and dynamics of defects in such structures were studied in detail in a previous work (Raad *et al.*, 2025 ▸).

In addition to its morphology, the position of the solid–liquid interface can be identified at each moment during the experiment. When a sample is pulled at a constant velocity during directional solidification, the solid–liquid interface initially undergoes a recoil within the imposed thermal field. This motion corresponds to the transition from rest to a steady-state position. For faceted interfaces, both the tip positions and the grooves between facets can be considered. However, measuring the grooves is significantly more complex and less accurate, since in most cases the groove ends are not visible. Given the heterogeneity of the tip positions, the interface evolution is tracked by following a line crossing as many visible tips as possible within the field-of-view. The recoil is defined as the difference between the instantaneous position and the initial position at rest. An example is shown in Fig. 2 ▸(*b*) (empty squares, right *y* axis) for *V*_p_ = 5 µm s^−1^ and *G*_0_ = 1.3 K mm^−1^. The recoil curve exhibits several distinct stages: an initial transient stage, during which the interface moves rapidly to its lowest position and then moves upward slower [gray shaded zone in Fig. 2 ▸(*b*)]; a second transient stage induced by the change in microscope objective; and finally, a steady-state stage, where the interface stabilizes within the non-regulated thermal zone. The initial objective is moved away from the non-thermalized area, the microscope turret is rotated to bring the second objective into position, and then the objective is moved closer to the sample, and the image is focused. This second objective has a much shorter working distance than the first, so although this process only takes a few tens of seconds, it affects the thermal field, inducing the second transient. Another example is shown in Fig. 3 ▸, from an experiment performed at a higher thermal gradient (*G*_0_ = 2.1 K mm^−1^), where no change in objective was made, and consequently the second transient was not observed [Fig. 3 ▸(*b*)]. In both cases, even after reaching a quasi-steady stable position, the interface exhibits fluctuations, which are mostly attributed to the dynamics of the tips.

The position of the interface relative to the melting temperature isotherm is directly linked to the undercooling at the interface. In the case of faceted interfaces, kinetic undercooling is dominant and plays an important role in determining the morphological stability of the interface. Furthermore, the relationship between the interface growth rate and kinetic undercooling defines different types of interface growth mechanisms as explained in the introduction (Flemings, 1974 ▸). By knowing the local thermal gradient and the interface recoil, the global interface undercooling can be estimated. The corresponding results for the previously discussed examples are presented in Figs. 2 ▸(*b*) and 3 ▸(*b*) (blue circles, left *y* axis). In Fig. 3 ▸(*b*), the undercooling and recoil curves are perfectly superimposed due to the constant local gradient within the non-regulated thermal zone. The rapid variation in undercooling observed during the transient stages corresponds to the interface transition from rest (where its velocity is zero) to a stationary state imposed by the pulling velocity. This causes the interface to recoil within the thermal field. As this recoil occurs, the growth rate increases, the facets grow, and the kinetic undercooling of the interface increases concomitantly, as it depends on the interface velocity.

**Figure 4:**
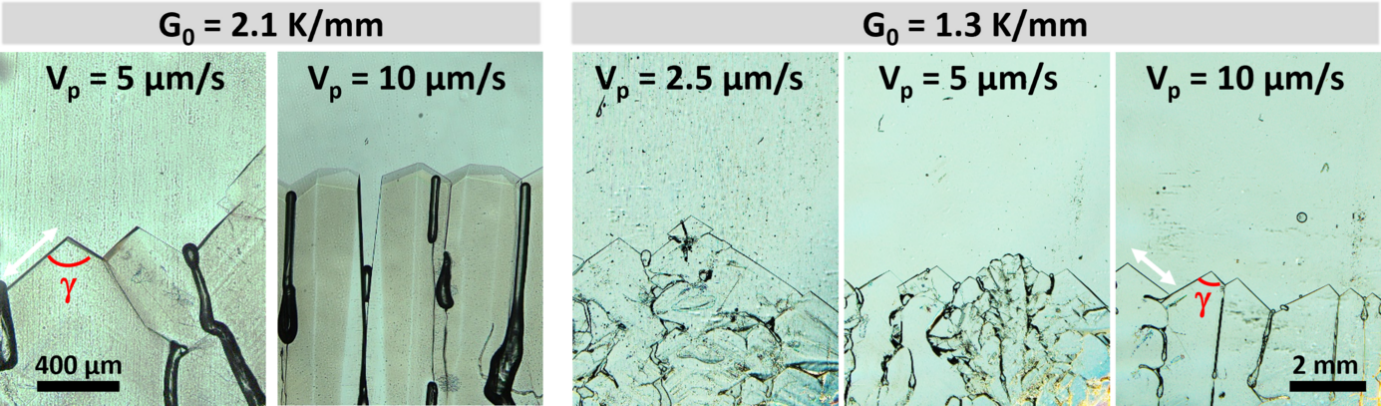
Salol solid–liquid interface morphology at steady-state for different experimental conditions at the same solidified length (40 mm). The white arrows exemplify the facet size, and γ, in red, represent the angle between the edges of facets.

The recoil and undercooling behaviors observed over time are consistent across all experiments. The steady-state results are compared between experiments in Table 1 ▸. Fig. 4 ▸ shows the respective interface morphologies. Note that the first two images have higher magnification compared to the last three images in the sequence. In addition to allow the determination of the undercooling, the interface recoil curves are also used to define the time period during which quantitative measurements are performed. For the following analysis, all quantities are measured exclusively during periods when the interface exhibits quasi-stationary behavior.

### 3.2. Facet characterization and crystallographic indexing

In the previous section, the interface was considered as a whole. However, given the polycrystalline nature of the sample, it is more insightful to examine each facet individually. Facets are the flat, planar surfaces that define the boundaries of a crystal during growth. These typically correspond to the slower-growing crystallographic planes, resulting in distinct, well defined flat surfaces. The *in situ* experiments performed in this study allow the observation and tracking of the two-dimensional projections of salol crystallographic planes. The edges of the facets are the most clearly distinguishable features. Since no preliminary orientation or crystallographic characterization is performed, the experimental images are analyzed to identify the crystallographic nature of the observed facets.

**Figure 5:**
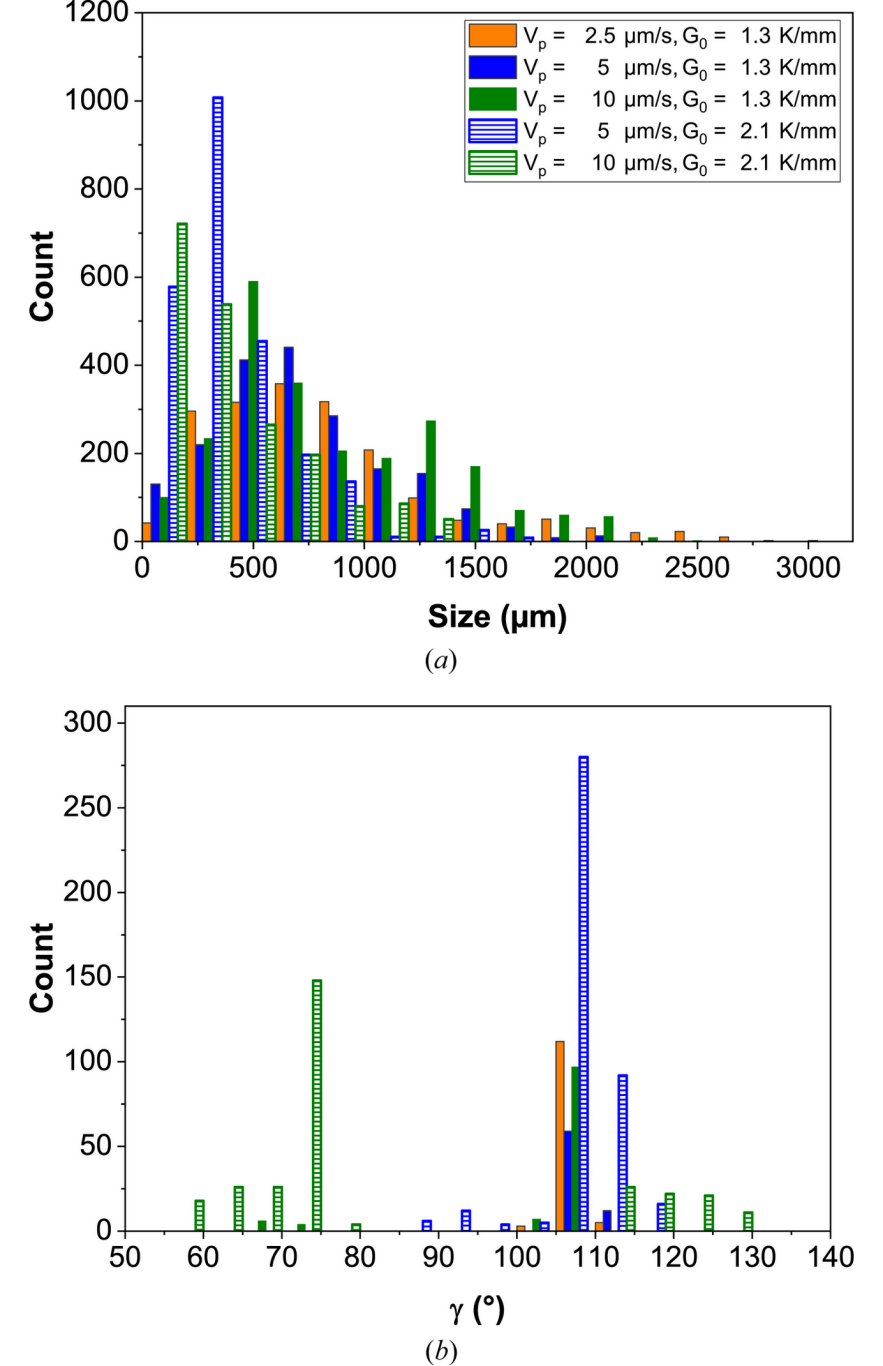
Distribution of (*a*) facet sizes and (*b*) projected angles between edges (γ), as function of pulling velocity for two thermal gradients.

Facet size refers to the dimensions of these planar surfaces. Several factors influence facet size, including pulling velocity, thermal gradient, crystallographic orientation, and defect occurrence during the solidification process (Podolinski, 1979 ▸; Stamelou *et al.*, 2017 ▸). In this study, facet size is defined as the edge length, as illustrated by the white arrows in Fig. 4 ▸. The measured facet sizes are highly heterogeneous. To further analyze this behavior, several facets were followed under all experimental conditions. Fig. 5 ▸(*a*) summarizes the results obtained for different pulling velocities at the two thermal gradients.

A reduction in the thermal gradient corresponds to an increase in facet size, as also reported in the literature (Lan & Tu, 2001 ▸). These authors found that the facet size is inversely proportional to the square root of the thermal gradient. This relationship, however, is not observed in the present experiments, where a gradient 1.2 times higher results in a facet size 2.2 times smaller. In contrast to previous studies (Shangguan & Hunt, 1991 ▸; Dey & Sekhar, 1993*a* ▸; Fabietti & Trivedi, 1997*a* ▸), an effect of pulling velocity is not clearly established here. The facet sizes obtained are of the same order of magnitude as those reported by Dey & Sekhar (1993*a* ▸) and are consistent with the distances between tips observed by Shangguan & Hunt (1991 ▸).

The quantitative differences observed with respect to the literature may be attributed to the polycrystalline nature of the samples in this study, as well as to the fact that a different sample was used for each experiment. Growth competition during the solidification process can promote the development of irregular facets, leading to statistical variations in facet sizes.

The interplanar angle is the angle formed between two neighboring crystallographic planes, which is equivalent to the angle between their normal vectors. It is a key parameter in crystallography, as it enables the crystallographic indexing of facets (Malgrange *et al.*, 2011 ▸; Millot & Nièpce, 2014 ▸). Salol exhibits a highly faceted solid–liquid interface, where each facet corresponds to a specific crystallographic plane. In the experimental images, it is not possible to measure interplanar angles directly. Instead, what can be measured is the angle between the edges of different facets, denoted by γ in Fig. 4 ▸. Thus, rather than determining directly the angle between plane normals, the angles between the directions corresponding to the facet edges, projected in two-dimensions, are measured.

These angles are measured for different facets during growth, and the results are shown in Fig. 5 ▸(*b*). Despite significant variability, two main peaks can be observed: a dominant one at 107.5° and another around 72.5°. These values agree with previous studies (Dey & Sekhar, 1993*a* ▸; Shangguan & Hunt, 1991 ▸). Considering the typical morphology of salol crystals with an orthorhombic structure (Neuroth & Klapper, 1994 ▸; Raad *et al.*, 2025 ▸; Wyrouboff, 1889 ▸) and the main crystal faces ({100}, {010}, {111}, {210} and {211}), the experimentally measured angles do not correspond to any expected edge directions. Therefore, a strategy is developed to identify the crystallographic nature of the observed facets.

**Figure 6:**
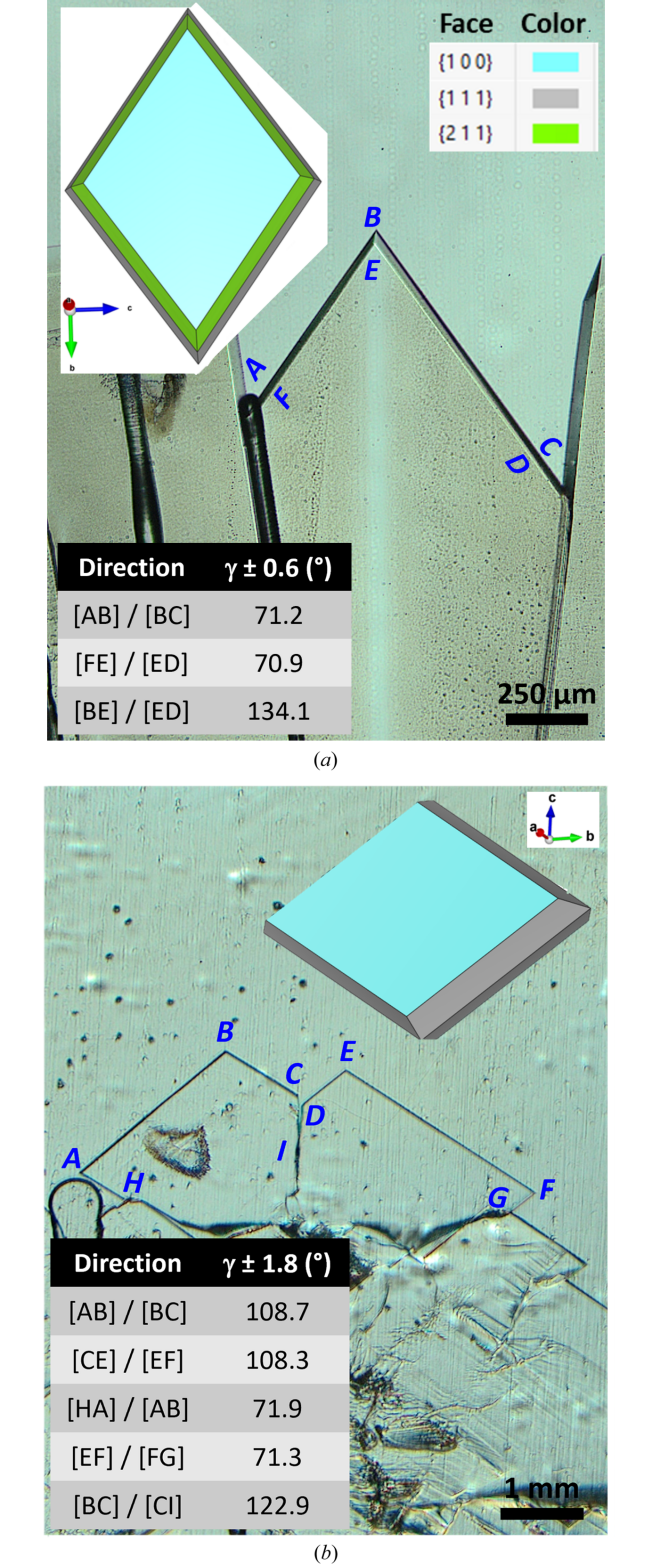
Salol solid–liquid interface with measured facet edge angles and the corresponding *VESTA*-generated crystal shapes: (*a*) *V*_p_ = 10 µm s^−1^, *G*_0_ = 2.1 K mm^−1^, *t* = 114 min; (*b*) *V*_p_ = 2.5 µm s^−1^, *G*_0_ = 1.3 K mm^−1^, *t* = 231 min.

For each faceted tip observed experimentally, the crystal shape generated in *VESTA* is rotated until it matches the experimental view. Once a satisfactory alignment is found, the corresponding orientation matrix is extracted. This matrix defines how the crystal axes (*a*, *b*, *c*) are oriented relative to the visualization coordinate system. Using the lattice parameters and applying the orientation matrix from *VESTA*, the Miller indices can be converted into Cartesian coordinates, allowing the calculation of both the real three-dimensional angle between two directions and its two-dimensional projection as observed experimentally. For a sample thickness of 50 µm and working distances around 5 mm, there is no significant difference between the projected angles obtained under orthogonal and perspective projections. Two representative examples are presented in Fig. 6 ▸, corresponding to the two dominant peaks in the histogram shown in Fig. 5 ▸(*b*).

Despite finding no significant effect of pulling velocity on the apex angles, it is noteworthy that facets with the lowest angles (65°–75°) are observed only at the higher pulling velocity (10 µm s^−1^). The faceted cell shown in Fig. 6 ▸(*a*) could be followed during its solidification over a longer duration than most other faceted cells, and the peak observed at 72.5° corresponds to its growth or to cells originating from its splitting. Points A to F are considered, and the angles between the corresponding segments are measured, as shown in the inset table of Fig. 6 ▸(*a*). In this crystal, the planes belonging to {010} and {210} are not visible, most likely due to the relatively high pulling velocity which favors bounding by denser planes. Because the longest edges of the {111} and {211} planes are parallel to the [011] direction, measuring only the apex angles ([AB]/[BC] and [FE]/[ED]) is not enough to determine whether both, or only one, of these planes are present in the experimental view. For this reason, the angle between [BE] and [ED] is also measured. The *VESTA*-generated salol crystal shape that best aligns with the experimental view is shown in the top inset of Fig. 6 ▸(*a*). The real angle between [011] and [011] directions is 70.53°, and the corresponding projected angle is 71.36° after applying the orientation matrix. The experimentally measured angles between [AB]/[BC] and [FE]/[ED] agree well with those expected between two [011] and [011] directions. The projected angles between [011] and [120] directions and between [011] and [110] directions are 140.93° and 135.79°, respectively. This indicates that the measured angle between [BE] and [ED] corresponds to the angle between [011] and [110] directions. Consequently, the facet defined by ABEF can be indexed as the (111) crystallographic plane, while the one defined by BCDE corresponds to the (111) plane. The {211} planes are not visible in this configuration. The large facet growing parallel, or almost, to the crucible wall corresponds to the (100) crystallographic plane.

Since the dominant peak in the histogram of Fig. 5 ▸(*b*) is at 107.5°, an example corresponding to facets of this type is presented in Fig. 6 ▸(*b*). In this case, two faceted tips are growing simultaneously, and the *VESTA*-generated salol crystal shape shown in the top inset aligns well with both tips. The experimentally measured angles between [AB]/[BC] and [CE]/[EF] agree well with those expected between the [011] and [011] directions after applying the orientation matrix (107.69°). Similarly, the angles measured between [HA]/[AB] and [FE]/[FG] correspond to 72.31°. The real 3D angles between [011] directions are 109.47° and 70.53°, respectively. Consequently, the facets defined by AB and CE can be indexed as the (111) crystallographic planes, while those defined by BC and EF correspond to the (111) planes. The angle between [BC] and [CI] is additionally measured, and it corresponds to an angle between [011] and [001] directions. The coincidence plane between the two faceted tips is the (010) crystallographic plane.

This same approach is applied to all growing facets to determine the real three-dimensional angles between the facet edges. As noted, the example shown in Fig. 6 ▸(*a*) is the only one where the real angle is 70.53°. For all other measurements, the angle is 109.47°. The main difference between the corresponding *VESTA*-generated crystal shapes in Fig. 6 ▸ is the growth direction. In Fig. 6 ▸(*a*), the growth is almost aligned with the *b* direction, while in Fig. 6 ▸(*b*), it is aligned with the *c* direction. Despite the initially random orientations and the polycrystalline nature of the samples, the *in situ* observations, without *post*-*mortem* analysis, indicate that the growth interface of salol is bounded by various combinations of {111} planes.

Dey & Sekhar (1993*a* ▸) also reported that the interface is consistently bounded by {111} planes when undercooling exceeds 3 K. They observed that as the growth velocity increases, undercooling also increases, influencing the selection of specific {111} planes. When growth conditions change, a transition in the facet angles occurs, with the system minimizing these angles by shifting from larger to smaller values. Interestingly, in the present study, a crystal growing with a tip angle of 70.5° was observed only at the higher pulling velocity and higher temperature gradient. However, given the polycrystalline nature of the samples, it is difficult to definitively attribute this behavior to the effect of pulling velocity.

### 3.3. Facet dynamics and growing laws

**Figure 7:**
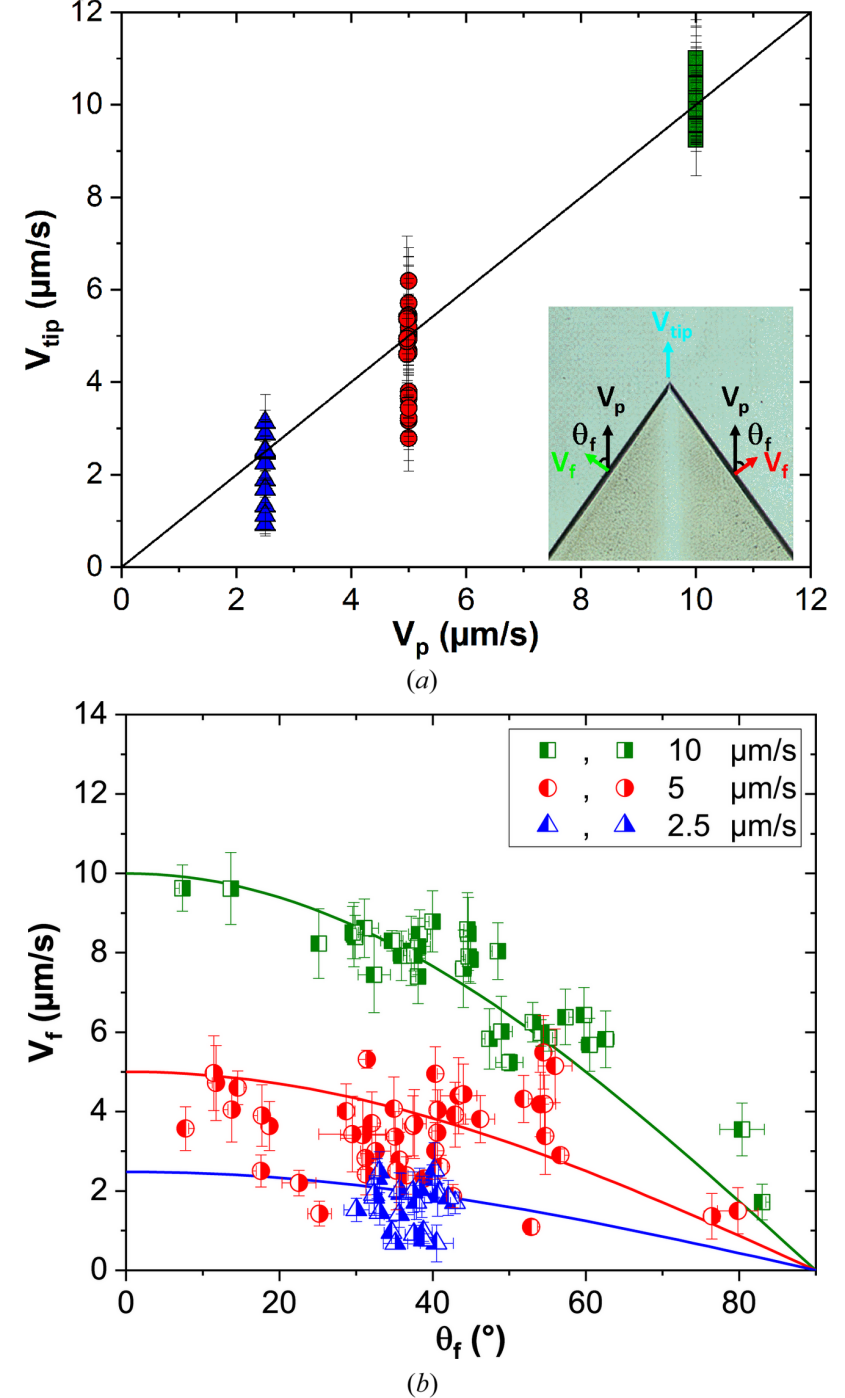
(*a*) Tip velocities (*V*_tip_) and (*b*) facet-normal growth velocities (*V*_f_) as functions of (*a*) pulling velocity (*V*_p_) and (*b*) facet orientation relative to the pulling direction (θ_f_), for all studied facets.

During directional solidification, the average speed at which the solid–liquid interface advances corresponds to the applied pulling velocity after the transient stage. However, the velocities of the faceted tips and facet itself are measured locally and instantaneously, and can be influenced by several factors (Jackson, 2010 ▸). Understanding the velocity of a faceted tip (*V*_tip_) is essential, as it reveals the dynamic behavior of the interface and helps identify the conditions that optimize the material growth. In general, the tips face upwards and grow in a direction close to the overall growth direction. Fig. 7 ▸(*a*) shows the tip velocities as function of the pulling velocity for all the studied faceted tips. Some dispersion is observed, and the measured velocities do not always align with the imposed pulling velocity. This mismatch originates from the presence of defects and growth competition processes. For example, at a pulling velocity of 5 µm s^−1^, the lowest tip velocity (2.6 µm s^−1^) is observed in a faceted tip that, although initially growing in a steady-state, is eventually eliminated due to growth competition. In contrast, a tip velocity higher than the imposed velocity usually corresponds to a newly formed faceted tip that is still accelerating.

During faceted growth, it is particularly important to consider the growth velocity in the direction normal to the facet (*V*_f_) (Dash *et al.*, 1999 ▸). The facet velocity, relative to its orientation with respect to the pulling direction (θ_f_), provides valuable data for understanding facet behavior. Under stationary conditions, the facet velocity corresponds to the projection of the pulling velocity onto the normal direction of the facet: *V*_f_ = *V*_P_cosθ_f_ [see inset of Fig. 7 ▸(*a*)]. Fig. 7 ▸(*b*) shows the measured facet velocities as a function of orientation for each facet, along with curves corresponding to stationary growth for different pulling velocities. In this figure, the full size of each symbol indicates the side of the faceted tip: the left-filled symbol corresponds to the (111) crystallographic planes, while the right-filled symbol represents the (111) planes. While the experimental data generally follow a downward trend similar to the theoretical curves, some points deviate significantly. At a pulling velocity of 10 µm s^−1^, one of the crystals axes (*a*, *b*, *c*) tends to align more closely with the pulling (thermal) direction, meaning that the facet orientations are more consistent and can be tracked throughout the solidification process. In contrast, at lower pulling velocities (2.5 and 5 µm s^−1^), the facet orientations are more random and it is harder to track the same facets over time. These deviations highlight the competitive nature of facet evolution during directional solidification, where local interactions and competition between facets significant influence the growth process. At higher pulling velocities, the system reaches a steadier state, while at the lower pulling velocities, the more dynamic and variable factors prevent the system from strictly adhering to steady-state predictions, leading to greater randomness. Factors such as stress accumulation, overgrowth by neighboring crystals, differences in undercooling between adjacent facets, growth striations, and bubble formation lead to local oscillations in facet velocity (Raad *et al.*, 2025 ▸). These effects contribute to the observed dispersion in the facet velocities.

**Figure 8:**
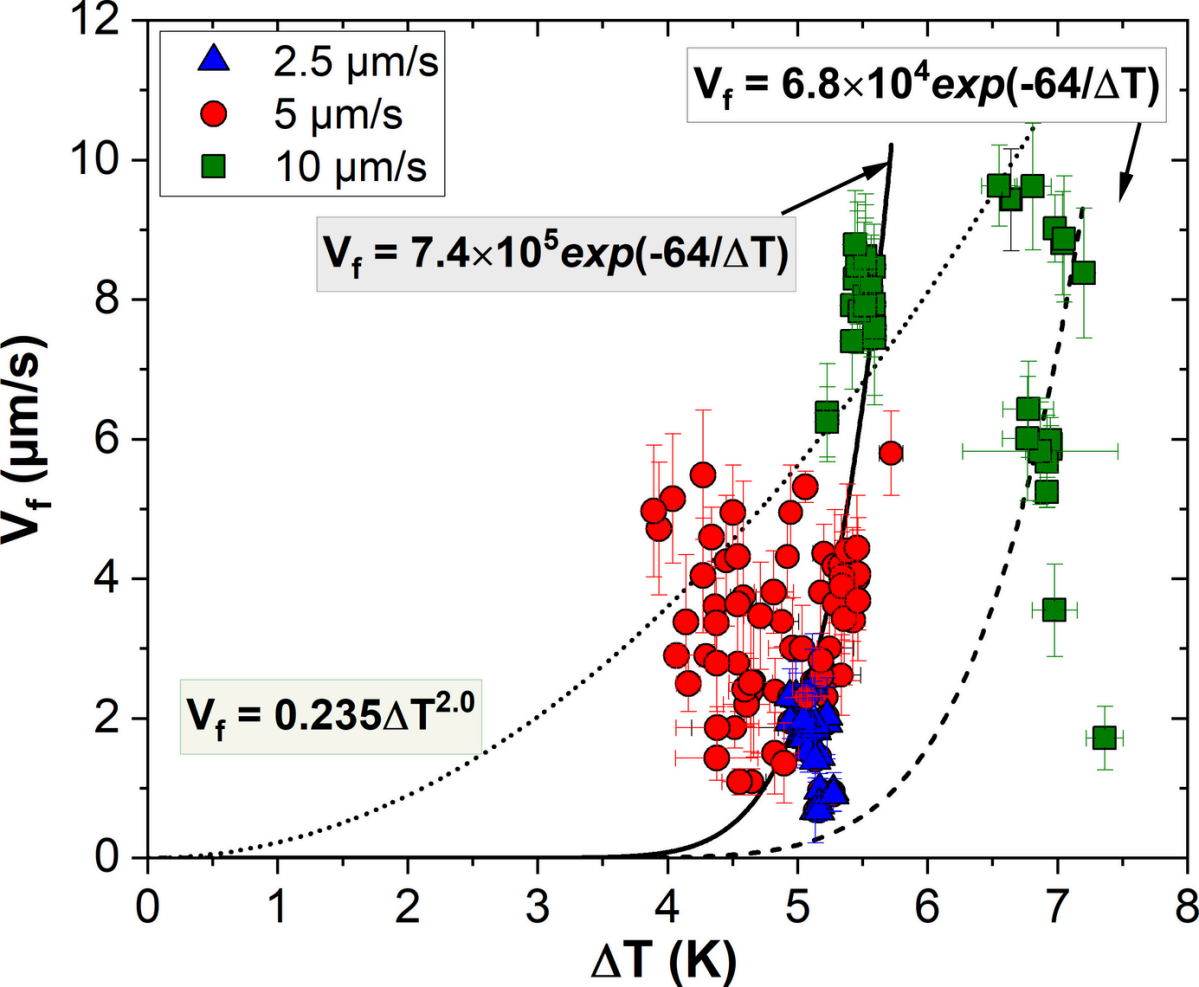
Measured normal facet velocities (*V*_f_) as a function of undercooling (Δ*T*) for all studied facets, compared with different kinetic law fits.

Finally, the facet velocities are analyzed as a function of undercooling in order to establish possible kinetic laws and identify the dominant growth mechanisms. In pure faceted materials, kinetic undercooling is generally expected to play a major role. Fig. 8 ▸ shows the facet velocities plotted as a function of undercooling, where the undercooling is measured at the tip. This measurement is considered more meaningful than one taken at the center of the facet, given the pronounced heterogeneity in facet sizes. The data are grouped by pulling velocity using the same color code as in Fig. 7 ▸. However, it is worth noting that all facets were previously identified as belonging to the {111} crystallographic family. Despite the significant scatter in the data, three fitting curves are proposed. The solid and dashed curves correspond to exponential laws characteristic of two-dimensional nucleation-controlled growth, while the dotted curve represents a power-law dependence typically associated with screw-dislocation-controlled growth. The majority of the experimental data are well described by the exponential law represented by the solid curve. This behavior is consistent with growth by two-dimensional nucleation, in which critical nuclei form on the crystal surface and subsequently expand to create new layers that cover the entire interface. This growth mode has previously been reported for salol crystals (Danilov & Malkin, 1954 ▸; Ie & Strickland-Constable, 1974 ▸; Jin *et al.*, 1990 ▸; Malkin, 1954 ▸; Morris *et al.*, 1968 ▸). It is important to emphasize that both the pre-exponential factor and the exponential coefficient are treated as free fitting parameters. Even under these conditions, a single exponential law cannot account for the full set of experimental data. A second exponential law of the same form, but with a lower kinetic prefactor, is therefore proposed. This law describes well the data obtained at the highest undercooling, corresponding to the experiment performed at *V*_p_ = 10 µm s^−1^ and *G*_0_ = 2.1 K mm^−1^, where the faceted tips are bounded by the 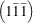 and 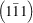 planes, associated with an interplanar angle of approximately 71°, in contrast to the other experiments where the facets correspond to (111) and (111) planes with interplanar angles close to 109°. The lower kinetic prefactor *A* indicates a less favorable nucleation process, suggesting that the solid seed orientation in this experiment promotes the formation of facets with smaller interplanar angles. Importantly, the exponential coefficients are similar for both laws, which reinforces the conclusion that the facets belong to the same crystallographic family. This coefficient corresponds to an effective energy barrier associated with two-dimensional nucleation and depends on the interfacial free energy γ, the melting enthalpy Δ*H*, and the mass per unit of area of the respective crystallographic plane ρ_2D_ (Burton *et al.*, 1951 ▸; Fabiyi *et al.*, 2022 ▸; Hillig & Turnbull, 1956 ▸):
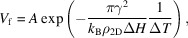
where *k*_B_ is the Boltzmann constant. Using literature values of Δ*H* = 19 kJ mol^−1^ and γ = 2.3 × 10^−2^ J m^−2^ (Wang & Inatomi, 2010 ▸; Kuribayashi *et al.*, 1996 ▸), the theoretical coefficient is estimated to be 67 K for the {111} crystallographic planes, in very good agreement with the experimentally obtained value of 64 K.

Several studies report that, at smaller undercoolings, salol growth may be dominated by the introduction of step sources (Ie & Strickland-Constable, 1974 ▸; Jin *et al.*, 1990 ▸; Neumann & Micus, 1954 ▸; Pollatschek, 1929 ▸; Riveros, 1968 ▸). Consistently, a limited number of data points, namely those obtained at *V*_p_ = 5 µm s^−1^ and *G*_0_ = 2.1 K mm^−1^, are also compatible with a power-law dependence. While such behavior is commonly associated with screw-dislocation-controlled growth, the limited number of points and the experimental scatter prevent a definitive conclusion. These results therefore suggest that two-dimensional nucleation is the dominant growth mechanism under most conditions, while screw-dislocation-controlled growth cannot be excluded under specific circumstances. A coexistence of both mechanisms, or a transition between them with increasing undercooling, remains a plausible interpretation.

## 4. Conclusion

This work presents a comprehensive quantitative investigation of the dynamic growth of the faceted solid–liquid interface of salol during directional solidification. Experiments are conducted under varying conditions to analyze the behavior of faceted interfaces. Through these experiments, factor influencing facet behavior, growth, and orientation are explored. The experiments begin with solid seeds of random and unknown orientation. The study reveals that the solid–liquid interface position stabilizes after a transient regime, despite fluctuations due to the dynamics of the faceted tips. The interface position relative to its rest position (the melting temperature for pure materials such salol) is directly linked to the undercooling at the interface, which in turn is related to the growth mechanism.

Several facets are monitored under all experimental conditions. The measured facet sizes are highly heterogeneous. An increase of the facet size is observed as the thermal gradient is increased. However, an effect of pulling velocity on facet size is not clearly established. It is also shown that growth competition during solidification promotes the development of irregular facets, causing irregular variations in facet size.

Although the samples initially have random orientations and are polycrystalline, the crystallographic orientation characterization reveals that the growth interface of salol is bounded by various combinations of {111} planes.

Understanding facet velocity is crucial, as it reveals the dynamic behavior of the interface and helps identify conditions that optimize material growth. Generally, the faceted tips grow in a direction close to the overall growth direction, although some dispersion is observed. Moreover, the measured tip velocities do not always align with the imposed pulling velocity. These mismatches arise from defects and growth competition processes, highlighting the influence of local interactions and dynamic events rather than strict adherence to steady-state predictions.

Finally, facet velocities are analyzed as a function of undercooling in order to establish possible kinetic laws and identify the dominant growth mechanisms. The majority of the experimental data are well described by an exponential law, characteristic of growth controlled by two-dimensional nucleation, in which critical nuclei form on the crystal surface and subsequently expand to cover the entire interface. The characteristic parameter of this law, corresponding to the exponential coefficient, is consistent across the different experimental conditions and agrees well with the theoretical value expected for salol. This parameter represents an effective barrier associated with two-dimensional nucleation and depends primarily on the interfacial free energy. Overall, these results indicate that two-dimensional nucleation is the dominant mechanism governing facet growth in salol under the present experimental conditions.

This works enhances the understanding of faceted solid–liquid interfaces in organic transparent materials, particularly salol. These findings underscore the complex interplay between solidification parameters and crystal morphology, providing valuable insights into crystal growth. The methodologies developed here offer a robust framework for further investigations and have potential applications in studying other systems.

## Supplementary Material

Crystal structure: contains datablock(s) I. DOI: 10.1107/S2052520626001769/tq5031sup1.cif

Structure factors: contains datablock(s) I. DOI: 10.1107/S2052520626001769/tq5031Isup2.hkl

CCDC reference: 2531769

## Figures and Tables

**Table 1:** Interface recoil (Δ*z*) and respective undercooling (Δ*T*) at steady state in different experiments

*G* (K mm^−1^)	*V*_p_ (µm s^−1^)	Δ*z* (mm)	Δ*T* (K)
2.1	5	2.3 ± 0.2	4.5 ± 0.3
2.1	10	3.5 ± 0.1	6.7 ± 0.2
1.3	2.5	9.0 ± 0.6	5.1 ± 0.1
1.3	5	8.3 ± 0.3	5.4 ± 0.1
1.3	10	7.7 ± 0.2	5.5 ± 0.1

## Data Availability

The authors declare that the data supporting the findings of this study are available within the paper and its supporting information files.
